# Notes on Preparations for the Sick

**Published:** 1892-12

**Authors:** 


### Notes on Preparations for the Sick.

---

**Liquor Sedans.**—PARKE, DAVIS & CO., Detroit.

This preparation, combining the properties of black haw, golden-seal, and Jamaica dogwood, is recommended as a utero-ovarian sedative and anodyne. Drugs of this kind appear to be gaining what is being lost by the various mechanical or surgical procedures; at any rate, these drugs are well worthy of trial before commencing more active surgical treatment, which may sometimes be rendered unnecessary. The preparation is an elegant one, and should be much appreciated by the medical gynæcologist who is not yet prepared to adopt the devices of his surgical confrère.

---

**Kola Malt.**—T. CHRISTY & CO., London.

This is an elegant preparation, combining the now well-known properties of the kola nut with the nutritious and digestive attributes of malt. Half a wineglassful, either alone or with water, cannot fail to be of service to invalids and convalescents.

**Ready made Linseed Poultices and Mustard Poultices (Sacklins' Patent).**—COOPER, 20 Royal Exchange, London.

The former are real poultices of pure crushed linseed rendered antiseptic. They are ready for immediate use on being soaked with boiling water. The latter are made of pure brown mustard flour and ground mustard husk. They only require to be moistened with warm water, and they can be worn by adults for hours. The benefit derived from a prolonged application of these comfortable poultices is likely to be much greater than from an application which stings so furiously that it is torn off in a few minutes.

---

Through Mr. H. T. Griffiths, of Victoria Street, Clifton, we have received various preparations, issued by MESSRS. BURROUGHS, WELLCOME & CO., London.

The evidence in favour of the utility of guaiacol in phthisis is steadily accumulating, and the effect appears to vary in proportion to the dose the patient is able to take. Most patients take it readily, and in one way or another the dose may be steadily increased up to 60 or 90 grains daily. In consequence of the supposed difficulties in the administration of guaiacol and creasote, attempts to find an efficient and more palatable substitute have led to the discovery of a tasteless compound of pure guaiacol with benzoic acid, which has already given good results in doses of twelve grains or more daily. This drug, to which the name of **Benzosol** has been given seems likely to have a brilliant future.

**Iodopyrin** is antipyrine in which an atom of hydrogen is replaced by iodine. When given internally it is split up into iodine and antipyrine, and the antipyretic value of the latter is in no degree impaired. It has yet to be proved whether the new drug presents any advantages over the older and more familiar antipyretics, such as phenacetin, antifebrine, and antipyrine.

The **Nasal Tabloids** are most convenient for nasal irrigation. Alkaline ones are prepared, each of which contains five grains of boric acid with five grains of sodium chloride, and is intended to be dissolved in a wineglassful of tepid water. There are also antiseptic ones with half a grain of carbolic acid and five grains of bicarbonate of soda and of powdered boric acid in each.

The **Naso-Pharyngeal Tabloid** contains common salt, boric acid, borax, benzoate of soda, cocaine, menthol, thymol, and oil of gaultheria. It must be somewhat difficult to ascertain the resultant effect of such a combination, but it should have a considerable range of utility.

The **Compressed Lithia Compound Tabloids,** prepared from the formula of Mr. Hugh Lane, contains salicylate of quinine (gr. $\frac{1}{3}$), with precipitated sulphur (grs. ij.) and benzoate of lithia (grs. iij.).

**Malto-Ricine** (Castor Oil and Malt Extract, KEPLER) is a palatable and effective combination, containing 50 per cent. of castor oil. The taste of the oil is so masked that even children will take it readily. The aperient action of the oil is decidedly enhanced by the intimate coalescence of the globules with the malt extract.

**Glycerinum Pepticum** (FAIRCHILD) is a concentrated solution of pepsine, being a glycerine extract of gastric mucous membrane, and containing no alcohol or other antiseptics than glycerine. It is uniform and permanent, freely miscible without precipitation, and so active that twelve minims will completely convert 2,500 grains of finely comminuted coagulated egg albumin into soluble albumoses and peptones. The dose is five to fifteen drops in acidulated water.

**Essentia Malti** (KEPLER) is described as "the ideal nutritive beverage," superior to stout, inasmuch as the carbo-hydrates and the diastase have not been sacrificed for the production of alcohol. A wineglassful of the Kepler essence contains more nutritive material than a pint of the finest alimental stout. Its flavour is good, and it makes an excellent drink when diluted with aerated water or milk.

